# Evaluation of Microbiological and Free-Living Protozoa Contamination in Dental Unit Waterlines

**DOI:** 10.3390/ijerph16152648

**Published:** 2019-07-24

**Authors:** Anna Maria Spagnolo, Marina Sartini, David Di Cave, Beatrice Casini, Benedetta Tuvo, Maria Luisa Cristina

**Affiliations:** 1 Department of Health Sciences, University of Genova, Via Pastore 1, 16132 Genova, Italy; 2 Department of Clinical Sciences and Translational Medicine, University of Rome Tor Vergata, 00133 Rome, Italy; 3 Department of Translational Research and New Technologies in Medicine and Surgery, University of Pisa, 56126 Pisa, Italy

**Keywords:** contamination, dental waterlines, infection risk

## Abstract

Studies conducted over the last 40 years have demonstrated that the water output from dental unit waterlines (DUWLs) is often contaminated with high densities of microorganisms. It has been monitored the microbiological quality of the water in 30 public dental facilities in northern Italy in order to assess the health risk for patients and dental staff. In each facility, samples of water both from taps and from DUWLs were analyzed in order to evaluate heterotrophic plate counts (HPCs) at 22 °C and 36 °C, and to detect coliform bacteria, *Pseudomonas aeruginosa*, *Legionella pneumophila* and amoebae. In 100% of the samples taken from the DUWLs, the concentration of HPCs was above the threshold as determined by the Ministère de la Santé et des Solidarités (2007). The concentration of *P. aeruginosa* was greater than the indicated threshold in 16.67% of the hand-pieces analyzed. A total of 78.33% of samples were contaminated by *L. pneumophila*, while in the samples taken from the DUWLs alone, this percentage rose to 86.67%. Amoebae were detected in 60% of the samples taken from hand-pieces; all belonging to the species *V. vermiformis*. This study documented the presence of various microorganisms, including *Legionella* spp., at considerably higher concentrations in water samples from DUWLs than in samples of tap water in the same facilities, confirming the role of the internal DUWLs in increasing microbial contamination, especially in the absence of proper management of waterborne health risks.

## 1. Introduction

Studies conducted over the last 40 years have revealed that the water output from dental unit waterlines (DUWLs) (i.e., narrow-bore plastic tubing that carries water to the high-speed hand-piece, air/water syringe and ultrasonic scaler) is often contaminated with high densities of microorganisms; [1,2] such contamination may be caused by the water supply itself, or, more probably, by the suck-back of biological fluids from the oral cavities of patients (back-contamination) [3,4].

Contamination levels ranging from 10^2^ to 10^8^ colony-forming units (CFU)/mL have regularly been reported [1,4,5]. These counts can occur because the features of dental unit waterlines (e.g., flow rates, materials and system design) promote both bacterial growth and the development of biofilm [2], which protects the organisms from desiccation, chemical aggression and predation [6].

The water conduit can be composed of approximately 6 m of narrow-bore flexible polyurethane or polyvinyl chloride (PVC) plastic tubing (1/16 in. or 2 mm diameter); narrow-bore tubing presents a very large ratio of surface area to volume (6:1), which encourages biofilm formation [7].

Moreover, water stagnation in DUWLs, when dental chair units (DCUs) are not in use, further encourages the growth of biofilm. Most DCUs are unlikely to be used for more than 12 h per day, 5 days per week, and thus water stagnation is a significant contributory factor to DUWL output water contamination [8].

DUWL contamination is associated with a wide variety of microorganisms, such as bacteria, fungi and viruses [4]. The range of microorganisms includes both environmental organisms (e.g., *Moraxella* spp. and *Flavobacterium* spp.) and opportunistic and true human pathogens (e.g., *Pseudomonas aeruginosa*, *Legionella pneumophila*, *Mycobacterium* spp., and *Staphylococcus* spp., etc.) [5,9,10,11]; this is of concern because of the increased risk of cross-infection, especially in immuno-compromised patients [7].

In addition to bacteria, fungi and viruses, protozoa such as free-living amoebae have also been isolated from dental unit waterlines. These protozoa may act as a reservoir for microorganisms (e.g., *Legionella* spp. *Pseudomonas* spp., etc.) or as pathogens themselves [12].

The concentration of amoebae intensifies around microbial biofilms and has been reported to be up to 300 times higher in DUWL output water than in tap water from the same source [13]. Protozoa isolated from DUWLs to date include Acanthamoeba, Giardia, Naegleria, Vannella, Vermamoeba, Vahlkampfia and Platyamoeba [13,14,15].

Contaminated dental unit water can pose an infection risk to both patients and dental practitioners.

Indeed, aerosols generated during dental procedures are a major source of the transmission of *Legionella* spp. and other bacterial pathogens in dental practice [16,17]; increased levels of Legionella antibodies in dental staff suggest that they may experience subclinical infection or mild Pontiac fever upon continuous exposure to contaminated aerosols [18]. To date, however, only in a small number of cases have there been reports of a proven link between local or systemic infections and exposure to contaminated DUWL. In one such case, an 82-year-old Italian woman died of pneumonia due to *Legionella* spp. following exposure to a dental unit contaminated by *L. pneumophila* serogroup 1. Molecular typing confirmed the clonal relationship between the clinical and environmental strains [19].

Although only a few cases of infection associated with contaminated DUWL output water have been reported, it is conceivable that such infections have not been identified because of the failure to associate infections with exposure to DUWL output water and aerosol [18].

We monitored the microbiological and free-living protozoa contamination of water from various public dental units in order to evaluate the health risk to patients.

## 2. Materials and Methods

### 2.1. Site Location

The investigation was carried out in 30 dental units located in two hospital facilities (A and B: 9 and 10 dental units, respectively) and in one Healthcare Utility (C: 11 units) in northern Italy. All the DUWLs were supplied directly by the municipal water network and no additional disinfection systems were used in the facilities.

In dental units examined, the activities were carried out only in the morning for 5–6 h, and the mean number of patients treated per day was 48, 51 and 60, in facilities A, B and C, respectively. None of the cubicles were equipped with a ventilation system. The dental units examined had been operating for many years (from 10 to >30).

Only in the dental units of facility A the internal waterlines were occasionally disinfected with H_2_O_2_; flow through hand-pieces for 30″ were performed at the start of day and after each patient only in the facility A.

### 2.2. Water Sampling and Microbiological Analysis

In each dental cubicle, a sample of water was taken both from the tap and from hand-pieces of the dental unit at the end of the dental activities; the tap water sample was used as a control to verify the quality of the water supplied to the building in which the unit was located. Specifically, 1 L of tap water and 1 L of dental unit water from hand-pieces was collected for the detection of *Legionella pneumophila*, and 250 mL was taken for the determination of heterotrophic plate counts (HPCs) at 22 °C and 36 °C, coliform bacteria and *Pseudomonas aeruginosa.* During sampling, it was also measured the temperature of the cold water from the washbasins in the cubicles, a sampling point taken to be representative of the water supplied to the dental unit.

Another liter of water was collected from the same devices for the detection of free-living protozoa (FLA) according to international standards [20].

The samples for microbiological analysis were collected in sterile plastic bottles, and sodium thiosulphate solution was added to the samples to neutralize free chlorine. The samples were immediately transported in portable coolers (at 4 °C) to the laboratory for chemical and microbiological analysis, and were processed within 24 h.

Analysis of the samples for Legionella was performed as described by ISO 11731-2:2004 [21]. Water samples were previously concentrated 100-fold by filtration through a 0.2 µm pore-size membrane (Millipore, Billerica, MA, USA). Serogrouping was performed by means of an agglutination test (Legionella latex test; Oxoid, Basingstoke, UK).

The results were expressed as colony-forming units (CFU) per liter. According to this method, the lower limit of detection is 100 CFU/L.

HPCs at 22 °C and 36 °C were determined in duplicate by means of the pour-plate method using ISO 6222 [22]. The total number of colonies was reported as CFU per milliliter.

Detection of coliform bacteria and *Pseudomonas aeruginosa* was performed by filtering 100 mL of water through 0.45 µm cellulose filters (Millipore, Billerica, MA, USA). The membranes were laid on Endo Agar Les plates (Liofilchem, Roseto degli Abruzzi (Te), Italy) and Cetrimide Agar (Oxoid, Basingstoke, UK) according to ISO 9308-1:2014 [23] for coliform growth and ISO 16266:2006 [24] for *Pseudomonas aeruginosa* growth. Species confirmation of suspect colonies was obtained by means of Mini API galleries (bioMeriéux, Marcy-l’Étoile, France). The results were expressed as CFU per 100 mL.

For amoeba detection was performed by the method described by Montalbano Di Filippo et al. [25]. One L samples were filtered through a 0.2 µm membrane (Millipore, Billerica, MA, USA) to search for free-living protozoa. The membrane filters were minced in 10 mL of sterile phosphate-buffered saline solution (PBS), pH 7.2, homogenized by vortex for 5 min and centrifuged at 1200× *g* for 15 min; 200 μL of pellet was inoculated onto non-nutrient agar (NNA) with a lawn of heat-inactivated *Escherichia coli* in Page’s amoeba saline solution (PAS), and incubated at 37 °C and were observed daily for amoebic growth up to 21 days. The presence of FLA was investigated by examining the NNA culture plates by inverted microscopy, using 20× and 40× objectives.

From all positive samples, the growing amoebae were harvested from culture plates, placed in Eppendorf tubes and washed twice with PBS, pH 7.4, before molecular analysis. DNA extraction was performed by means of the QIAamp DNA Micro Kit (Qiagen, Milan, Italy). To identify FLA species, 18S rDNA amplification with primers P-FLA-F and P-FLA-R was performed. This PCR allows the simultaneous amplification of FLA, amplicon lengths varying from 500 to 1500 base pairs (*V. vermiformis*: 800 bp; *N. fowleri*: 900 bp; *Vannella* sp., *Vahlkampfiaovis*: 950 bp; *Acanthamoeba* species: from 1080 to 1500 bp) as described by Tsvetkova et al. 2004 [26]. All PCR amplification reactions were performed in a 50 μL mixture containing 25 μL PCR master mix 2X (Promega, Milan, Italy), 6 μL template DNA, and 20 pmol of each primer in a TProfessional Basic Thermocycler (Biometra GmbH, Göttingen, Germany). The PCR products were visualized by means of electrophoresis on 1% agarose gel stained by SYBR Safe DNA gel stain (Invitrogen, Monza (MB), Italy). PCR amplicons were purified by using a mi-PCR Purification Kit (Metabion GmbH, Steinkirchen, Germany) according to the manufacturer’s instructions, and directly sequenced on both strands by Bio-Fab Research (Rome, Italy). Sequences were edited by means of the FinchTV 1.4 software (Geospiza Inc., Seattle, WA, USA) and aligned by Clustal Omega. To assign the isolates to the right species level, phylogenetic analysis was performed in which the sequences obtained were compared with those of reference strains.

### 2.3. Statistical Analysis

Statistical analysis was carried out by means of STATA/SE^TM^ 14.2 software (Stata Corp LP, College Station, TX, USA). The results were analyzed in terms of descriptive statistics, and differences between groups were evaluated by means of a non-parametric Wilcoxon rank-sum test and a Pearson’s Chi-square test. The relationship between variables was estimated by applying the Spearman’s correlation test. A *p* value < 0.05 was considered statistically significant.

## 3. Results

Microbiological analysis of the water, both from the taps and from the dental units, did not reveal the presence of coliform bacteria.

Table 1 reports the concentrations of HPCs at 22 °C and 36 °C, *L. pneumophila* and *P. aeruginosa* in hand-pieces and tap water.

These concentrations were higher in water samples from the hand-pieces than in the tap water with regard to all the microbiological parameters investigated, though statistically significant differences emerged only for HPCs at 22 °C and 36 °C (*p* < 0.001).

In tap water, on comparing the values of the HPCs at 22 °C and 36 °C and of *P. aeruginosa* with the target values (≤100 CFU/mL, ≤10 CFU/mL, and <1 CFU/100 mL, respectively, as determined by the Ministère de la Santé et des Solidarités in 2007 [27], a high percentage of nonconformity was recorded for HPCs, while no sample proved positive for *P. aeruginosa*.

**Figure 1:**
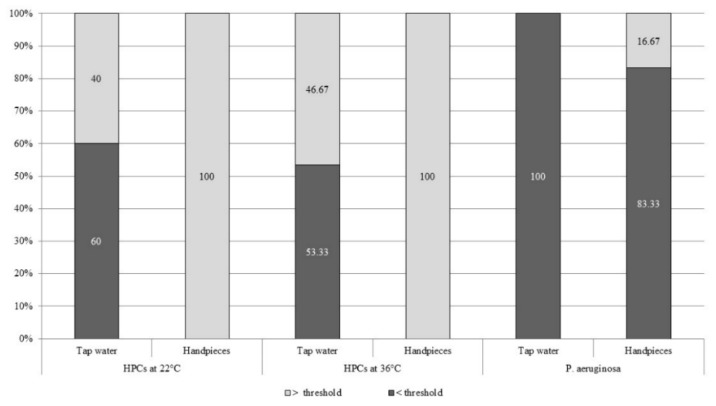
Percentage distribution of samples above and below target values for HPCs at 22 °C (100 CFU/mL or less), HPCs at 36 °C (10 CFU/mL or less), and *P. aeruginosa* (<1 CFU/100 mL) in tap water and hand-piece water.

Similarly, we determined the percentage of water samples from hand-pieces that were above/below the target levels of the above-mentioned parameters (Figure 1).

*L. pneumophila* was detected in 47 samples of water (78.33%): 21 samples of tap water and 26 samples from hand-pieces. A concentration of ≥10^3^ CFU/L was found in 26.67% of samples from dental units, while it no sample of tap water was this concentration exceeded. The presence of *L. pneumophila* sg 1 was of 23.40%.

Amoebae were detected in 60% of water samples from hand-pieces and in 23.33% of tap water samples. Their presence was significantly higher in water from dental units than in tap water (*p* <0.01).

**Figure 2:**
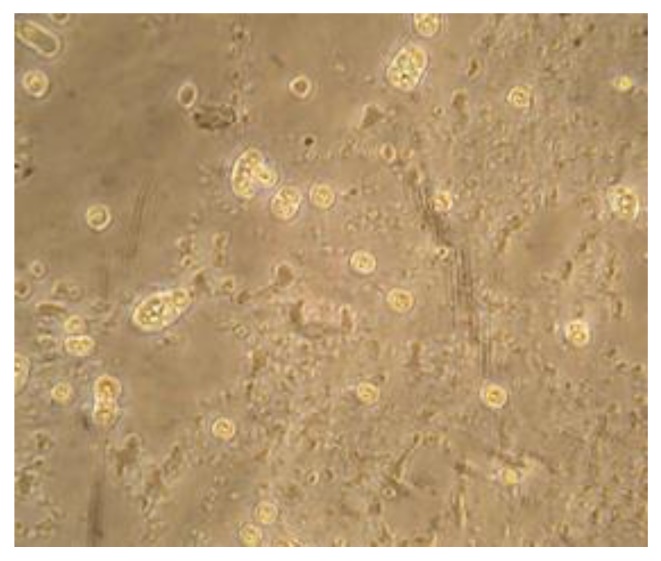
*Vermamoeba vermiformis* from hand-piece water samples (inverted microscope, 40× objective).

All PCR positive isolates showed bands with an approximate size of 800 bp (expected for *Vermoamoeba vermiformis*). The analyses unambiguously identified all samples as *Vermoamoeba vermiformis* (100%), the sequences showing the highest identity (99%) with those accessible in GenBank. No water sample analyzed in this study was characterized by the presence of other FLA species. Figure 2 shows *Vermamoeba vermiformis* from water samples from hand-pieces.

Table 2 shows the mean concentrations of *L. pneumophila* and *P. aeruginosa* in the presence and absence of amoebae.

A significantly higher concentration of *L. pneumophila* was detected in the samples of hand-piece water positive for amoebae than in amoeba-negative samples (*p* < 0.05).

Regarding tap water, no statistically significant differences in the concentrations of *L. pneumophila* emerged in relation to the presence/absence of amoebae.

It was also evaluated the possible correlation between amoebae and both *L. pneumophila* and *P. aeruginosa*; an analysis conducted by means of the Spearman correlation test revealed a statistically significant positive correlation between the presence of amoebae and *L. pneumophila* (rho = 0.2961, *p* < 0.05).

Regarding the tap water temperature, the mean value was 21 °C ± 2.3 °C.

## 4. Discussion

The microbial contamination of DUWLs varies according to several factors: The age and the model of the dental unit, the presence/absence of anti-backflow valves (which prevent back-contamination), the adoption of control measures (e.g., flushing), the methods of disinfection used, the features of the water supply system, etc. DUWLs supplied directly by the municipal water network may be more frequently contaminated, particularly in large distribution systems (as in hospitals), where the large volume of water storage tanks and the sections with reduced water circulation provide optimal conditions for the growth of Legionella and other waterborne pathogens [28].

With regard to the characteristics of the dental units examined in the present study, all the units were supplied directly by the municipal water network, and none of the buildings in which the units were located was equipped with a system for the disinfection of piped water. Only in the dental units of one hospital facility the internal waterlines were occasionally disinfected with H_2_O_2_; however, no disinfection protocol indicating the concentration of H_2_O_2_ to be used or the frequency and duration of disinfection was implemented.

The guidelines issued by the Centers for Disease Control and Prevention (CDC) on infection control in dental health care settings recommend that level of HPCs in dental unit output water should not exceed 500 CFU/mL [2]. The American Dental Association (ADA) has set a heterotrophic bacteria load of ≤200 CFU/mL for water delivered from dental unit waterlines [29]. In the EU, there is no current guideline for DUWLs, but in some countries reference is made to the drinking water standard [7]. In Italy, no national limit values have been placed on HPCs (at 22 °C and 36 °C), nor on drinking water or the water used in healthcare facilities. Consequently, we took the values determined by the Ministère de la Santé et des Solidarités in 2007 as target values (≤100 CFU/mL, ≤10 CFU/mL, respectively) [27]. Likewise, with regard to *P. aeruginosa*, we considered a target value of <1 CFU/100mL.

In the dental units examined, the concentration of HPCs (at 22 °C and 36 °C) exceeded the indicated threshold in 100% of the samples analyzed, while in tap water the target values were exceeded in less than 50% of the samples. Moreover, the mean concentrations of HPCs at 22 °C and 36 °C in the water delivered from hand-pieces proved to be far higher (1168.53 ± 906.00 CFU/mL and 827.90 ± 746.87 CFU/mL, respectively) than the target values, with concentrations of up to 3200 CFU/mL and 2116 CFU/mL, respectively.

Such a high microbial load may constitute a serious risk of infection, since: (a) Patients requiring dental treatment may be immuno-compromised; (b) the treatment may be invasive, thus introducing potential pathogens into the circulation; (c) the water is aerosolized and may contaminate both surfaces and the air, thereby increasing the risk of infecting both patients and dental staff [30].

The mean concentration of HPCs in the water in the dental units proved to be significantly higher (*p* < 0.001) than in tap water.

Regarding the concentration of *P. aeruginosa*, this was seen to exceed the indicated threshold in 16.67% of hand-pieces analyzed, with mean values of 25.13 ± 77.75 CFU/100 mL. By contrast, in no sample of tap water was this microorganism detected.

Various microorganisms present in dental units may have a considerable healthcare impact. One of these is Legionella, which is contracted through aspiration or inhalation of aerosolized contaminated water [31]. Indeed, contaminated water sources can spread spray or droplets containing Legionella, leaving airborne particles of less than 5 μm in diameter that can be deeply inhaled [32,33,34,35].

A variety of dental equipment, including high- and low-speed hand-pieces, ultrasonic instruments, and water spray syringes, aerosolize water from DUWLs [31]. The reported prevalence of Legionella in DUWLs varies widely: from 0 to 100% [36,37].

In our study, it emerged that, of the total number of water samples analyzed, 47/60 (78.33%) were contaminated by Legionella; on considering only the samples from the dental units, the percentage of positivity rose to 86.67%.

Previous studies have reported that *L. pneumophila* serogroup (sg) 1 is often present in the water supplies of facilities, including healthcare facilities [38]. In our study, sg 1 of *L. pneumophila* was detected in 23.40% of the water samples.

European guidelines [27] on the concentrations of *Legionella* spp. in healthcare facilities have set a target value (10^3^ CFU/L), an alert value (10^4^ CFU/L) and an action value (>10^4^ CFU/L). In 26.67% of the dental units that we examined, we detected a concentration of >10^3^ CFU/L, while in no sample was the concentration of 10^4^ CFU/L reached.

The mean concentration of *L. pneumophila* in the water from the dental units was 676.67 ± 746.34 CFU/L. However, it must be stressed that the concentration detected by means of a culture method could underestimate the real number of surviving bacterial cells, as *L. pneumophila* develops symbiotic relationships with protists such as amoebae [39]. Indeed, once inside the amoeba cell, bacteria will survive or escape the adverse conditions presented by digestive vacuoles; moreover, they can also find sanctuary from unfavorable environmental conditions and can multiply [40].

The association of Legionella with free-living amoebae was first reported in 1980 [41]. *L. pneumophila* has been shown to be able to parasitize and multiply in more than twenty different protozoan species, including Acanthamoeba, Naegleria and Vermamoeba [42,43].

*Vermamoeba vermiformis* is predominant in DUWLs, and probably enters these devices initially from fresh water supplies from storage tanks [11]. However, *Vermamoeba* species are also reported to have been isolated from the throats of humans as long ago as 1967 [44], implying that the *V. vermiformis* in DUWLs could also come from humans. In a study conducted by Michel R et al. [45], 215 water samples were taken from 49 dental treatment units and investigated for the presence of free-living amoebae. *V. vermiformis* was found in 10.6% of the samples.

In our study, 60% of the water samples from hand-pieces contained amoebae, and in significantly higher concentrations (*p* < 0.01) than tap water. All the amoebae proved to belong to the species *V. vermiformis*; moreover, the samples from hand-piece water that were positive for amoebae displayed a significantly higher concentration of *L. pneumophila* than amoeba-negative samples (*p* < 0.05). Moreover, the Spearman correlation test revealed a statistically significant positive correlation between the presence of amoebae and *L. pneumophila* (rho = 0.2961, *p* < 0.05).

With regard to the relationship between *P. aeruginosa* and *V. vermiformis*, the results of several studies have been controversial; in some studies, amoebae have fed effectively on *P. aeruginosa* [46,47], while in others this species has proved to be toxic [48,49,50]. In this regard, a study conducted by Delafont et al. [51] showed that *V. vermiformis* could be permissive for *P. aeruginosa*, depending on the bacterial strains and environmental conditions. The results of our study revealed a four-fold higher mean concentration of *P. aeruginosa* in water samples from hand-pieces that were negative for *V. vermiformis* than in amoeba-positive samples. The difference, however, did not prove statistically significant.

Data from the present study suggest the presence of considerable contamination in DUWLs that could be a source of hazards, not only for dental patients (especially immuno-compromised patients) but also for healthcare personnel, who are routinely exposed to the same risk [9]. The higher prevalence of antibodies to *L. pneumophila* in dentists and dental staff, in comparison with the general population, suggests a potential health risk for these workers [52]. Moreover, aerosols produced by turbines, micro-motors, air/water syringes and ultrasound scalers can contaminate both surfaces and the air. With regards to air contamination, ventilation systems can ensure adequate air exchange, thereby reducing the risk of infection. For what concerns surfaces, the control of transmission of infections in the healthcare setting involves regular disinfection of potentially contaminated surfaces [53].

## 5. Conclusions

The present study documented the presence of various microorganisms, including Legionella, at considerably higher concentrations in water samples from dental units than in samples of tap water in the same facilities.

Many guidelines suggest numerous approaches to be adopted in order to reduce DUWL contamination, including both non-chemical (including flushing, drying, and applying an antimicrobial filter) and chemical methods [2,54]. The application of chemical agents has been demonstrated to be more effective than non-chemical methods [55]. The CDC recommends that any devices that enter a patient’s mouth (e.g., hand-pieces, ultrasonic scalers, or air/water syringes) should be connected to the waterline and flushed for at least 20 s between patients [2].

The flushing of dental waterlines has been shown to reduce the levels of planktonic bacteria in the water, but this practice has not been shown to affect the biofilm that accumulates in the waterlines [56]. Among the chemicals now available, peracetic acid is one of the most powerful biocidal agents; it exerts a rapid, broad-spectrum biocidal activity and could be useful in controlling DUWL contamination. However, it has a series of side-effects, which have limited its use in dentistry [55]. Moreover, various studies have evaluated the synergistic effects of different types of methods, which have proved superior to those of single methods.

Moreover, it is important to educate dental staff on how to implement it. Simply treating waterlines may not be sufficient to ensure water quality; indeed, it is extremely important to verify the efficacy of the methods used through regular monitoring.

## Figures and Tables

**Table 1:** Concentrations of heterotrophic plate counts (HPCs) at 22 °C and 36 °C (colony-forming units (CFU)/mL), *L. pneumophila* (CFU/L) and *P. aeruginosa* (CFU/100 mL) in water from hand-pieces and tap water.

		Mean ± SD	Min–Max	Median	Interquartile Range	*p* Value
HPCs at 22 °C	Hand-piece	1168.53 ± 906.00	145–3200	881.5	548–1710	<0.001
	Tap water	385.27 ± 836.41	2–2880	37.5	15–200	
HPCs at 36 °C	Hand-piece	827.90 ± 746.87	27–2116	518.5	109–1620	<0.001
	Tap water	242.93 ± 241.64	2–1860	9.5	5–121	
*P. aeruginosa*	Hand-piece	25.13 ± 77.75	0–308	0	0–0	0.26
	Tap water	0	0–0	0	0–0	
*L. pneumophila*	Hand-piece	676.67 ± 746.34	0–2700	350	200–1300	0.15
	Tap water	343.33 ± 313.69	0–900	300	0–700	

**Table 2:** Concentrations of *L. pneumophila* (CFU/L) and *P. aeruginosa* (CFU/100 mL) in the presence and absence of amoebae.

			Mean ± SD	Min-Max	Median	Interquartile Range
*L. pneumophila*	Hand-pieces	No amoebae	333.33 ± 405.27	0–1400	200	100–400
		Amoebae	905.56 ± 839.80	0–2700	550	300–1500
	Tap water	No amoebae	321.74 ± 293.81	0–900	300	0–500
		Amoebae	414.29 ± 389.14	0–800	700	0–700
*P. aeruginosa*	Hand-pieces	No amoebae	46.50 ± 109.30	0–308	0	0–0
		Amoebae	10.89 ± 45.21	0–192	0	0–0

## References

[1] Fujita M., Mashima I., Nakazawa F. (2015). Monitoring the decontamination efficacy of the novel Poseidon-S disinfectant system in dental unit water lines. J. Microbiol. Immunol. Infect..

[2] Kohn W.G., Harte J.A., Malvitz D.M., Collins A.S., Cleveland J.L., Eklund K.J., Centers for Disease Control and Prevention (2004). Guidelines for infection control in dental health care settings-2003. J. Am. Dent. Assoc..

[3] Cristina M.L., Spagnolo A.M., Sartini M., Dallera M., Ottria G., Perdelli F., Orlando P. (2009). Investigation of organizational and hygiene features in dentistry: A pilot study. J. Prev. Med. Hyg..

[4] Costa D., Mercier A., Gravouil K., Lesobre J., Delafont V., Bousseau A., Verdon J., Imbert C. (2015). Pyrosequencing analysis of bacterial diversity in dental unit waterlines. Water Res..

[5] Walker J.T., Bradshaw D.J., Bennett A.M., Fulford M.R., Martin M.V., Marsh P.D. (2000). Microbial biofilm formation and contamination of dental-unit water systems in general dental practice. Appl. Environ. Microbiol..

[6] Leoni E., Dallolio L., Stagni F., Sanna T., D’Alessandro G., Piana G. (2015). Impact of a risk management plan on Legionella contamination of dental unit water. Int. J. Environ. Res. Public Health.

[7] Walker J.T., Marsh P.D. (2007). Microbial biofilm formation in DUWS and their control using disinfectants. J. Dent..

[8] O’Donnell M.J., Boyle M.A., Russell R.J., Coleman D.C. (2011). Management of dental unit waterline biofilms in the 21st century. Future Microbiol..

[9] Lizzadro J., Mazzotta M., Girolamini L., Dormi A., Pellati T., Cristino S. (2019). Comparison between Two Types of Dental Unit Waterlines: How Evaluation of Microbiological Contamination Can Support Risk Containment. Int. J. Environ. Res. Public Health.

[10] Costa D., Bossard V., Brunet K., Fradin B., Imbert C. (2017). Planktonic free-living amoebae susceptibility to dental unit waterlines disinfectants. Pathog. Dis..

[11] Lal S., Singhrao S.K., Achilles-Day U.E., Morton L.H., Pearce M., Crean S. (2015). Risk Assessment for the Spread of Serratia marcescens Within Dental-Unit Waterline Systems Using Vermamoeba vermiformis. Curr. Microbiol..

[12] Vanessa B., Virginie M., Nathalie Q., Marie-Hélène R., Christine I. (2012). Hartmannella vermiformis can promote proliferation of Candida spp. in tap-water. Water Res..

[13] Barbeau J., Buhler T. (2001). Biofilms augment the number of free-living amoebae in dental unit waterlines. Res. Microbiol..

[14] Dillon A., Achilles-Day U.E., Singhrao S.K., Pearce M., Morton L.H.G., Crean S. (2014). Biocide sensitivity of Vermamoeba vermiformis isolated from dental-unit-waterline systems. Int. Biodeterior. Biodegrad..

[15] Leduc A., Gravel S., Abikhzer J., Roy S., Barbeau J. (2012). Polymerase chain reaction detection of potentially pathogenic free-living amoebae in dental units. Can. J. Microbiol..

[16] Cristina M.L., Spagnolo A.M., Sartini M., Dallera M., Ottria G., Lombardi R., Perdelli F. (2008). Evaluation of the risk of infection through exposure to aerosols and spatters in dentistry. Am. J. Infect. Control..

[17] Perdelli F., Spagnolo A.M., Cristina M.L., Sartini M., Malcontenti R., Dallera M., Ottria G., Lombardi R., Orlando P. (2008). Evaluation of contamination by blood aerosols produced during various healthcare procedures. J. Hosp. Infect..

[18] Arvand M., Hack A. (2013). Microbial contamination of dental unit waterlines in dental practices in Hesse, Germany: A cross-sectional study. Eur. J. Microbiol. Immunol..

[19] Ricci M.L., Fontana S., Pinci F., Fiumana E., Pedna M.F., Farolfi P., Sabattini M.A., Scaturro M. (2012). Pneumonia associated with a dental unit waterline. Lancet.

[20] American Public Health Association (2005). Standard Methods for the Examination of Water and Wastewater.

[21] International Organization for Standardization (ISO) (2004). Water Quality—Detection and Enumeration of Legionella—Part 2: Direct Membrane Filtration Method for Waters with Low Bacterial Counts.

[22] International Organization for Standardization (ISO) (1999). Water Quality: Enumeration of Culturable Micro-Organisms, Colony Count by Inoculation in a Nutrient Agar Culture Medium.

[23] International Organization for Standardization (ISO) (2014). Water Quality—Enumeration of Escherichia Coli and Coliform Bacteria—Part 1: Membrane Filtration Method for Waters with Low Bacterial Background Flora.

[24] International Organization for Standardization (ISO) (2006). Water Quality—Detection and Enumeration of Pseudomonas Aeruginosa—Method by Membrane Filtration.

[25] Montalbano Di Filippo M., Santoro M., Lovreglio P., Monno R., Capolongo C., Calia C., Fumarola L., D’Alfonso R., Berrilli F., Di Cave D. (2015). Isolation and molecular characterization of free-living amoebae from different water sources in Italy. Int. J. Environ. Res. Public Health.

[26] Tsvetkova N., Schild M., Panaiotov S., Kurdova-Mintcheva R., Gottstein B., Walochnik J., Aspock H., Lucas M.S., Muller N. (2004). The identification of free-living environmental isolates of amoebae from Bulgaria. Parasitol. Res..

[27] Ministère de la Santé e des Solidarités (2007). L’eau Dans les Etablissements de Santé. http://nosobase.chu-lyon.fr/Reglementation/2005/guide_eau_etabs.pdf.

[28] Casini B., Buzzigoli A., Cristina M.L., Spagnolo A.M., Del Giudice P., Brusaferro S., Poscia A., Moscato U., Valentini P., Baggiani A. (2014). Long-term effects of hospital water network disinfection on Legionella and other waterborne bacteria in an Italian university hospital. Infect. Control Hosp. Epidemiol..

[29] American Dental Association (1996). ADA Statement on Dental unit waterlines. J. Am. Dent. Assoc..

[30] Orlando P., Cristina M.L., Dallera M., Ottria G., Vitale A., Badolati G. (2008). Surface disinfection: Evaluation of the efficacy of a nebulization system spraying hydrogen peroxide. J. Prev. Med. Hyg..

[31] Estrich C.G., Gruninger S.E., Lipman R.D. (2017). Rates and predictors of exposure to Legionella pneumophila in the United States among dental practitioners: 2002 through 2012. J. Am. Dent. Assoc..

[32] Montagna M.T., De Giglio O., Napoli C., Diella G., Rutigliano S., Agodi A., Auxilia F., Baldovin T., Bisetto F., Arnoldo L. (2018). Control and prevention measures for legionellosis in hospitals: A cross-sectional survey in Italy. Environ. Res..

[33] Montagna M.T., De Giglio O., Cristina M.L., Napoli C., Pacifico C., Agodi A., Baldovin T., Casini B., Coniglio M.A., D’Errico M.M. (2017). Evaluation of Legionella Air Contamination in Healthcare Facilities by Different Sampling Methods: An Italian Multicenter Study. Int. J. Environ. Res. Public Health.

[34] Montagna M.T., De Giglio O., Cristina M.L., Albertini R., Pasquarella C., Agodi A., Coniglio M.A., GISIO-SItI Working Group, AIA Working Group, SIMPIOS Working Group (2017). Legionella indoor air contamination in healthcare environments. SpringerBriefs in Public Health.

[35] Montagna M.T., De Giglio O., Napoli C., Cannova L., Cristina M.L., Deriu M.G., Delia S.A., Giuliano A., Guida M., Laganà P. (2014). Legionella spp. contamination in indoor air: Preliminary results of an Italian multicenter study. Epidemiol. Prev..

[36] Pankhurst C.L., Coulter W.A. (2007). Do contaminated dental unit waterlines pose a risk of infection?. J. Dent..

[37] Carinci F., Scapoli L., Contaldo M., Santoro R., Palmieri A., Pezzetti F., Lauritano D., Candotto V., Mucchi D., Baggi L. (2018). Colonization of Legionella spp. in dental unit waterlines. J. Biol. Regul. Homeost. Agents.

[38] Montagna M.T., Cristina M.L., De Giglio O., Spagnolo A.M., Napoli C., Cannova L., Deriu M.G., Delia S.A., Giuliano A., Guida M. (2016). Serological and molecular identification of Legionella spp. isolated from water and surrounding air samples in Italian healthcare facilities. Environ. Res..

[39] Dietersdorfer E., Cervero-Aragó S., Sommer R., Kirschner AK., Walochnik J. (2016). Optimized methods for Legionella pneumophila release from its Acanthamoeba hosts. BMC Microbiol..

[40] Marciano-Cabral F., Jamerson M., Kaneshiro E.S. (2010). Free-living amoebae, Legionella and Mycobacterium in tap water supplied by a municipal drinking water utility in the USA. J. Water Health.

[41] Rowbotham T.J. (1980). Preliminary report on the pathogenicity of *Legionella pneumophila* for freshwater and soil amoebae. J. Clin. Pathol..

[42] Spagnolo A.M., Orlando P., Perdelli F., Cristina M.L. (2016). Hospital water and prevention of waterborne infections. Rev. Med. Microbiol..

[43] Balczun C., Scheid P.L. (2017). Free-Living Amoebae as Hosts for and Vectors of Intracellular Microorganisms with Public Health Significance. Viruses.

[44] Wang S.S., Feldman H.A. (1967). Isolation of Hartmannella species from human throats. N. Engl. J. Med..

[45] Michel R., Just H.M. (1984). Acanthamoebae, Naegleria and other free-living Amoebae in cooling and rinsing water of dental treatment units. Zentralbl. Bakteriol. Mikrobiol. Hyg. B.

[46] Rogerson A., Berger J. (1981). Effect of crude oil and petroleum-degrading micro-organisms on the growth of freshwater and soil protozoa. Microbiology.

[47] Weitere M., Bergfeld T., Scott S.A., Matz C., Kjelleberg S. (2005). Grazing resistance of Pseudomonas aeruginosa biofilms depends on type of protective mechanism, developmental stage and protozoan feeding mode. Environ. Microbiol..

[48] Groscop J.A., Brent M.M. (1964). The effects of selected strains of pigmented microorganisms on small free-living amoebae. Can. J. Microbiol..

[49] Qureshi M.N., Perez A.A., Madayag R.M., Bottone E.J. (1993). Inhibition of Acanthamoeba species by Pseudomonas aeruginosa: Rationale for their selective exclusion in corneal ulcers and contact lens care systems. J. Clin. Microbiol..

[50] Singh R.N. (1945). The selection of bacterial food by soil amoebae and the toxic effects of bacterial pigments and other products on soil protozoa. Br. J. Exp. Pathol..

[51] Delafont V., Rodier M.H., Maisonneuve E., Cateau E. (2018). Vermamoeba vermiformis: A Free-Living Amoeba of Interest. Microb. Ecol..

[52] Spagnolo A.M., Cristina M.L., Casini B., Perdelli F. (2013). Legionella pneumophila in healthcare facilities. Rev. Med. Microbiol..

[53] Perdelli F., Dallera M., Cristina M.L., Sartini M., Ottria G., Spagnolo A.M., Orlando P. (2008). A new microbiological problem in intensive care units: Environmental contamination by MRSA with reduced susceptibility to glycopeptides. Int. J. Hyg. Environ. Health.

[54] Ministero della Salute (2015). Linee Guida Per la Prevenzione ed il Controllo Della Legionellosi.

[55] Montebugnoli L., Dolci G. (2002). A new chemical formulation for control of dental unit water line contamination: An in vitro and clinical study. BMC Oral Health.

[56] Rice E.W., Rich W.K., Johnson C.H., Lye D.J. (2006). The role of flushing dental water lines for the removal of microbial contaminants. Public Health Rep..

